# Supplementary material for: Complex‐centric proteome profiling by SEC‐SWATH‐MS
Source: Mol Syst Biol. 2019 Jan 14;15(1):e8438. doi: 10.15252/msb.20188438 (PMC6346213; doi:10.15252/msb.20188438)
Supplement: Supplementary file 8 — Dataset EV7 [file MSB-15-e8438-s008.zip › feature_plots_string/P13667.pdf]

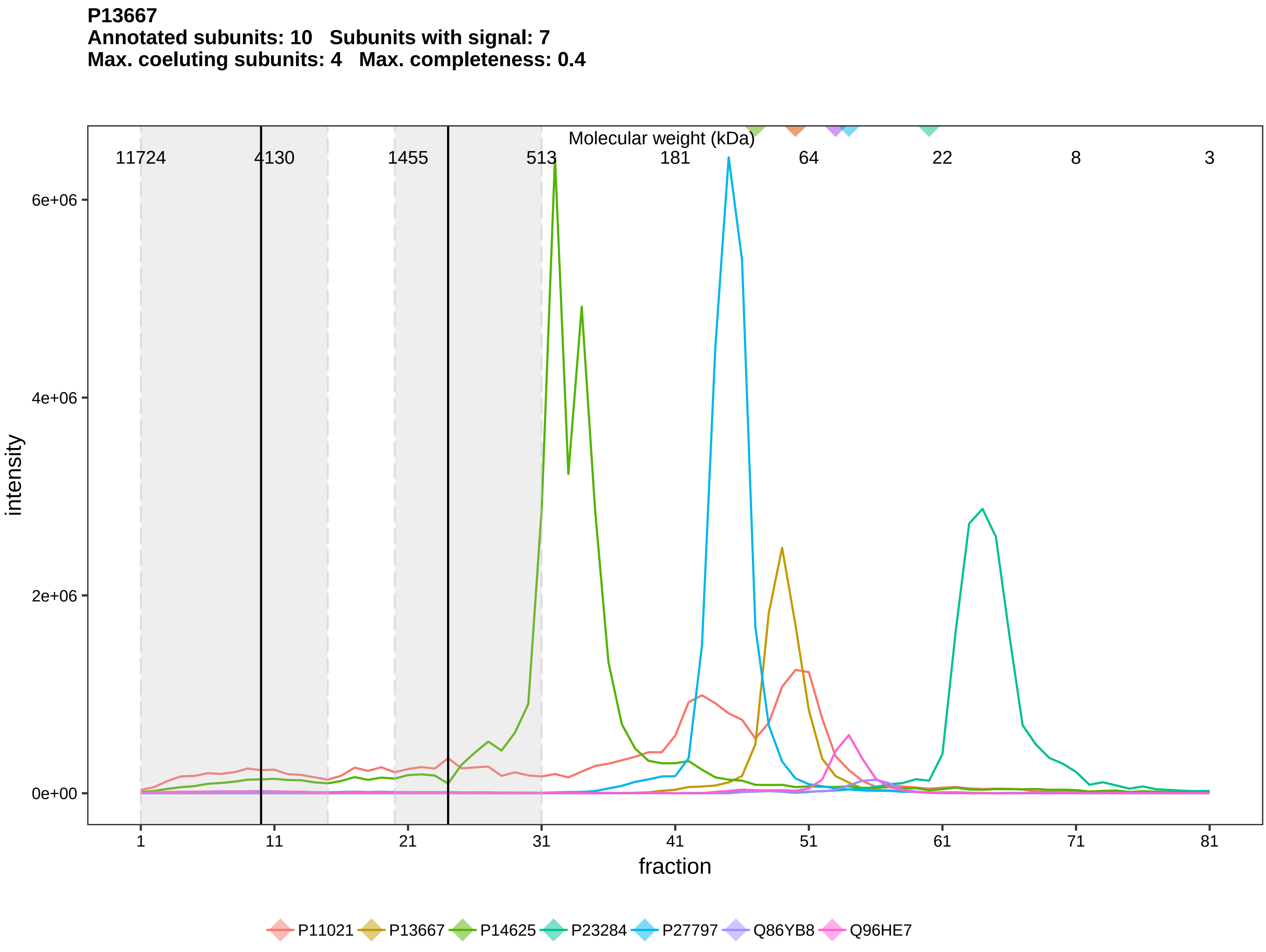

P13667
Annotated subunits: 10 Subunits with signal: 7
Max. coeluting subunits: 4 Max. completeness: 0.4
Molecular weight (kDa)
11724
4130
1455
513
181
64
22
8
3
6e+06
4e+06
2e+06
0e+00
intensity
1
11
21
31
41
51
61
71
81
fraction
P11021
P13667
P14625
P23284
P27797
Q86YB8
Q96HE7
